# Liquid Biopsy-Based Biomarkers of Inflammatory Nociception Identified in Male Rats

**DOI:** 10.3389/fphar.2022.893828

**Published:** 2022-06-27

**Authors:** Christina R. Merritt, Irma E. Cisneros, Obdulia Covarrubias-Zambrano, Sonja J. Stutz, Massoud Motamedi, Stefan H. Bossmann, Kathryn A. Cunningham

**Affiliations:** ^1^ Center for Addiction Research, University of Texas Medical Branch, Galveston, TX, United States; ^2^ Department of Pharmacology and Toxicology, University of Texas Medical Branch, Galveston, TX, United States; ^3^ Department of Pathology, University of Texas Medical Branch, Galveston, TX, United States; ^4^ Department of Chemistry, Kansas State University, Manhattan, KS, United States; ^5^ Department of Cancer Biology, The University of Kansas Cancer Center, Kansas City, MO, United States; ^6^ Department of Ophthalmology and Visual Sciences, University of Texas Medical Branch, Galveston, TX, United States

**Keywords:** liquid biopsy, biomarker, λ-carrageenan, inflammatory pain, CXCL1, CCL2, MMP9, MMP2

## Abstract

Physicians are challenged in treating pain patients due to the lack of quantifiable, objective methods of measuring pain in the clinic; pain sensation is multifaceted and subjective to each individual. There is a critical need for point-of-care quantification of accessible biomarkers to provide objective analyses beyond the subjective pain scales currently employed in clinical care settings. In the present study, we employed an animal model to test the hypothesis that circulating regulators of the inflammatory response directly associate with an objective behavioral response to inflammatory pain. Upon induction of localized paw inflammation, we measured the systemic protein expression of cytokines, and activity levels of matrix metalloproteinases (MMPs) that are known to participate in the inflammatory response at the site of injury and investigated their relationship to the behavioral response across a 24 h period. Intraplantar injection with 1% λ-carrageenan induced a significant increase in paw thickness across this timespan with maximal effects observed at the 8 h timepoint when locomotor activity was also impaired. Expression of the chemokines C-X-C motif chemokine ligand 1 (CXCL1) and C-C motif chemokine ligand 2 (CCL2) positively correlated with paw inflammation and negatively correlated with locomotor activity at 8 h. The ratio of MMP9 to MMP2 activity negatively correlated with paw inflammation at the 8 h timepoint. We postulate that the CXCL1 and CCL2 as well as the ratio of MMP9 to MMP2 activity may serve as predictive biomarkers for the timecourse of inflammation-associated locomotor impairment. These data define opportunities for the future development of a point-of-care device to objectively quantify biomarkers for inflammatory pain states.

## Introduction

There is a drought in the development of medications for the treatment of acute and chronic pain. The National Institutes of Health report a mere 2% of candidate pain medications advance from Phase I clinical trials to FDA-approval (Thomas and Wessel, 2017), as opposed to 10% of medications for other conditions. Clinician scientists are challenged in identifying novel, effective medications for pain patients due to the lack of quantifiable, objective methods to measure pain in the clinic; pain sensation is multifaceted and subjective for each individual (Younger et al., 2009). The International Association for the Study of Pain defines pain as “an unpleasant sensory and emotional experience associated with actual or potential tissue damage” (Raja et al., 2020). Due the unavoidable subjectivity of the pain experience as a whole, diagnostic measurements in the clinic should instead focus on quantifying proteins involved in nociceptive inflammation that contribute to tissue repair and restoration processes. The current gold standard of pain measurement at the point-of-care (POC) relies on self-report measures such as the Numerical Rating Scale or Visual Analogue Scale. Self-report results can be distorted by such factors as emotional state, cultural background, and cognitive abilities (Krebs et al., 2007). Clinical studies that investigated the predictive relationship between self-report of pain and physical performance tasks (i.e., “Up and Down” test in osteoarthritis patients) within the same individual found a modest relationship between the two measures, with correlation coefficients of 0.3 or lower (Simmonds et al., 1998; Novy et al., 2002). Skin conductance and body temperature may be used as physiological indicators of pain (Harrison et al., 2006; Storm, 2008), however, the validity of their use has been challenged by inconsistent findings (Hu et al., 2019). Lastly, neuroimaging tools (e.g., functional magnetic resonance imaging) are utilized to identify a neurologic signature of physical pain in cortical regions involved in regulating pain sensation such as the insular cortex (Apkarian et al., 2005; Wager et al., 2011; Lee et al., 2021). While these imaging techniques can provide important information about pain processing, these tests are expensive, time intensive, and do not provide a quantitative measurement. There is a critical need for economical, rapid POC quantification of accessible inflammation biomarkers to provide objective analyses beyond the subjective pain scales currently employed in clinical care settings (Chizh and Hobson, 2007).

The concept of screening body fluids (e.g., blood, saliva, urine) for molecular biomarkers of disease (i.e., liquid biopsy) is not new (Mandel and Metais, 1948), however, recent technological advancements enable these approaches to become powerful and innovative diagnostic tools in the clinic (Mader and Pantel, 2017; Poulet et al., 2019). At present, several liquid biopsy assays are FDA-approved for early detection of circulating tumor cells or circulating tumor DNA using next-generation sequencing. For example, a recent study sought to determine the clinical validity of liquid biopsy screening in a cohort of 514 metastatic castration-resistant prostate cancer patients and found genomic aberrations related to prostate cancer in blood samples of 94% of the diagnosed patients (Sonpavde et al., 2019). This is one of many examples that demonstrate high concordance between positive liquid biopsy screening and the clinical diagnosis (for review) (Vaidyanathan et al., 2018). Additionally, liquid biopsies possess several advantages including minimal invasiveness, decreased risk, and importantly, the ability to sample repeatedly to monitor disease progression and treatment response. Liquid biopsy diagnostic tools for cancer biomarkers show great promise for clinical utility and are spurring interest in expanding application to different pathologies, including osteoarthritis and joint inflammatory diseases (Michela, 2021). Current methodologies for quantifying pain in the clinic are inadequate; the liquid biopsy approach would greatly benefit physicians seeking to accurately diagnose the etiology and severity of tissue inflammation, as well as treatment efficacy across time in patients experiencing nociceptive inflammatory pain.

The present preclinical study was designed to identify blood-born biomarkers that directly correlate with the timecourse and intensity of acute inflammatory nociception. In turn, these biomarkers could provide prioritized candidates for diagnostic testing in pain patients at the POC. To achieve this, we induced paw edema through subplantar λ-carrageenan injection (Otterness and Moore, 1988) and determined the temporal relationship between localized inflammation and expression of inflammatory mediators in rodent plasma. The xenobiotic λ-carrageenan is regularly used to induce an acute inflammation and behavioral hyperalgesia (Boyce-Rustay et al., 2010; Da Silva et al., 2012; Armbruster et al., 2017). Concurrently, we monitored for potential impairment of voluntary, spontaneous movement in a familiar environment as a non-reflexive, non-evoked surrogate measurement of inflammatory nociception (Zhu et al., 2012; Cho et al., 2013; Sheahan et al., 2017). Reduced mobility is a hallmark symptom in pain patients and thus is more translational in comparison to more commonly used preclinical nociceptive models, such as the Hargreaves radiant heat assay of thermal sensitivity (Hargreaves et al., 1988) or von Frey filament test of mechanical sensitivity (Chaplan et al., 1994). Our approach is to assess and establish the potential feasibility for detection of inflammation biomarkers at the POC in pain patients.

## The Biochemical Origin of Pain

The “Law of Pain,” introduced by Sota Omoigui, states that “the origin of all pain is inflammation and the inflammatory response” (Omoigui, 2007b;a). Initiation of inflammation and the nociceptive state occurs when basophils, mast cells, and platelets release nitric oxide (Cury et al., 2011), histamine (Obara et al., 2020), and serotonin (Sommer, 2004; Loyd et al., 2013) in immediate response to localized tissue damage. The combined cellular response to these stimuli increases local blood flow and leads eventually to the secretion of prostaglandins, which facilitate the attraction of specialized immune cells (e.g., macrophages) to initiate the inflammatory response. Macrophages release pro-inflammatory interleukins (IL) (e.g., IL-1β, IL-4, IL-6, IL-8), anti-inflammatory cytokines (e.g., IL-4, IL-10, IL-11, IL-13) and chemokine (C-C and C-X-C motif) ligands [e.g., C-X-C motif chemokine ligand 1 (CXCL1), C-C motif chemokine ligand 2 (CCL2)] (Hughes and Nibbs, 2018) that attract and activate other immune cells such as granulocytes (neutrophils, eosinophils), lymphocytes (T-helper cells), as well as fibroblasts and endothelial cells to the inflamed tissue. Using Bio-Plex technology, we screened plasma samples against a broad panel of these cytokines to determine the relationship of peripheral expression relative to the timecourse of acute inflammatory nociception.

The cocktail of proteases [e.g., granzyme B, matrix metalloproteinases (MMPs), neutrophil elastase, and many others], bactericidal peptides and proteins, that is, generated by immune cells upon injury leads inevitably to tissue damage and pain sensations (Raoof et al., 2018). The MMPs include collagenases (e.g., MMP-1, -8, -13), stromelysins (e.g., MMP-3, -10, -11), and especially gelatinases (e.g., MMP-2 and -9) which play important functions in acute and chronic inflammation (Lakhan and Avramut, 2012) and are regulated by the competition of post-translational proteolytic cleavage and endogenous inhibitors (Quintero-Fabian et al., 2019). In general, MMP-2 is constitutively expressed by numerous cell types, whereas MMP-9 is induced by tumor necrosis factor-α (TNF-α) and IL-1β released from inflammatory cells (neutrophils, macrophages, and lymphocytes) (Lakhan and Avramut, 2012). MMP-9 plays a major role in the migration of cells through the walls of blood vessels to inflammatory sites and degradation of collagen in the extracellular matrix to aid in tissue remodeling processes (Ahmed et al., 2011; Lakhan and Avramut, 2012; Ramirez et al., 2018). MMP-9 is transiently overexpressed and then activated during an early response to tissue damage, whereas MMP-2 is a signature enzyme of the late response. In contrast to virtually all previous studies, the nanobiosensor technology employed here permits the quantitative determination of proteolytic enzyme activities. This is of translational importance because MMPs require proteolytic activation (Lopez-Otin and Bond, 2008). Based on this paradigm, the quotient of MMP-9 divided by MMP-2 activity can be used as an indicator for the temporal course of inflammatory nociception (Kawasaki et al., 2008).

## Optical Nanobiosensors for Quantitative Determination of Matrix Metalloproteinase 2 and Matrix Metalloproteinase 9 Activities

Taking advantage of the progress in the field of applied nanotechnology, Bossmann and colleagues developed nanoparticle-based biosensors for the ultra-sensitive detection of proteases in liquid biopsies (Wang et al., 2014; Udukala et al., 2016; Malalasekera et al., 2017; Kalubowilage et al., 2018). The proteolytic activity of numerous MMPs can be detected at sub-femtomolar levels and across a wide detection range (10^−5^–10^−16^ M) (Wang et al., 2014; Udukala et al., 2016). The core of the detection nanoparticle is a dopamine-coated iron/iron oxide core/shell nanoparticle with a diameter of 15–17 nm, including a 1–2 mm Fe_3_O_4_ shell (Wang et al., 2014) which is linked to the two Foerster Resonance Energy Transfer (FRET) nanobiosensors including tetrakis-carboxyphenyl-porphyrin (TCPP) and cyanine 5.5 (Cy 5.5). Whereas Cy 5.5 is permanently tethered, TCPP is linked *via* a protease-cleavable consensus sequence. Once this oligopeptide is cleaved, TCPP escapes plasmonic quenching by the Fe core and FRET by Cy 5.5, leading to a measurable fluorescence increase which is detected by a conventional plate reader. Here, we report the application of Fe/Fe_3_O_4_-based nanobiosensor technology to detect circulating biomarkers related to the inflammatory nociceptive state (Wang et al., 2014). We selected MMP-2 and MMP-9 activities to assess in the plasma collected from our rat model for inflammatory nociception due to the important role these enzymes play in tissue repair and restoration processes (Leira et al., 2007; Bruschi et al., 2016; Medeiros et al., 2017; Hannocks et al., 2019; Escolano-Lozano et al., 2021).

We postulated that inflammation and locomotor impairment would peak 5–8 h following λ-carrageenan treatment and return to baseline levels at 24 h (Otterness and Moore, 1988; Lin et al., 2015; Annamalai and Thangam, 2017). We also hypothesized that variation in expression or activity of inflammatory regulators will be target-specific, as each plays a unique role in the inflammatory response (Zhang and An, 2007). To our knowledge, this study is the first to capture the full timecourse of protein expression and activity of selected inflammatory regulators in alignment with inflammatory nociception within subjects. A better understanding of the timecourse of these fluctuations will aid in predicting onset, duration, and termination of the pain sensation in patients and ultimately be valuable to enhance management of coinciding symptoms. These efforts align with a key aspect of the Helping to End Addiction Long-Term (HEAL™) initiative, launched by the National Institutes of Health in 2019: acceleration of the discovery and validation of biomarkers, endpoints, and signatures for pain conditions.

## Methods

### Animals

Male Sprague-Dawley rats (*n* = 20) were obtained with surgically implanted, jugular vein catheters fitted with a PinPort™ (Instech Laboratory, Plymouth Meeting, PA) implanted by the supplier (Envigo, Indy). Rats were acclimated to the colony room for 2 weeks prior to the start of the experiment and housed one/cage to prevent damage to external tubing of catheter. Food and water were available *ad libitum* and rats were handled daily throughout the entire study. Six rats were excluded from the experiment prior to baseline measurements due to loss of catheter patency. Two rats were statistical outliers in baseline measures of locomotor activity or cytokine expression and excluded from all analyses. Final analyses of all experiments include six rats per treatment. All experiments were carried out in accordance with the National Institutes of Health Guide for the Care and Use of Laboratory Animals (2011) and with approval from the University of Texas Medical Branch Institutional Animal Care and Use Committee.

## Experimental Procedures

**FIGURE 1:**
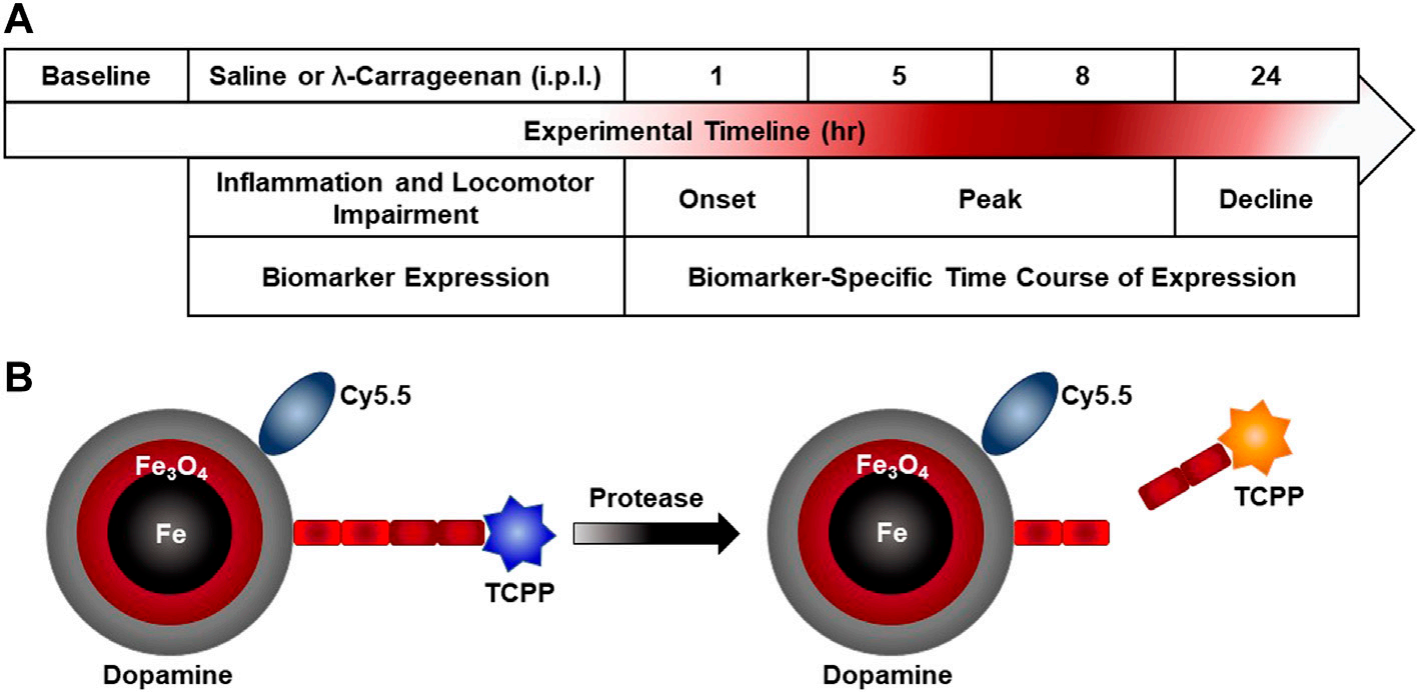
Experimental timeline and nanobiosensor schematic. **(A)** Paw inflammation, non-evoked inflammatory pain behavior, and biomarker expression were assessed prior to intraplantar (i.p.l.) treatment with saline or λ-carrageenan. The anticipated timeline of λ-carrageenan on these measures at 1–24 h following treatment is noted. **(B)** The schematic provides an overview of nanobiosensor technology for protease activity detection which consists of a dopamine-coated iron/iron oxide nanoparticle, amide-tethered cyanine 5.5 (Cy 5.5) and tetrakis-carboxyphenyl-porphyrin (TCPP), that is, attached *via* a consensus sequence. Upon addition of a protease (MMP-2 and MMP-9), the consensus sequence is cleaved and TCPP is released and subsequently switches on TCPP fluorescence.

Figure 1A illustrates the experimental timeline. Paw inflammation, non-evoked inflammatory nociceptive behavior, and biomarker expression were assessed prior to treatment with saline or λ-carrageenan. The anticipated timeline of λ-carrageenan on these measures at 1–24 h following treatment is noted.

### λ-Carrageenan-Induced Inflammation

Rats received an intraplantar (i.p.l.) 0.1 ml injection of saline (0.9% NaCl) or 1% λ-carrageenan dissolved in water (Type IV Lambda, Sigma-Aldrich; molecular weight = 579) to induce acute inflammation and swelling of the paw. Injections were administered into the footpad of the right hindpaw using a 26-gauge beveled needle. Vernier calipers (Scienceware, Bel-Art Products) were used to measure paw thickness (mm) as an indicator of paw inflammation. Calipers were aligned perpendicularly across the center of the footpad. Experimenters marked the center of the footpad prior to baseline measurement to ensure consistency across the timecourse.

### Plasma Collection

Blood (0.5 ml) was collected from PinPort™ rats and transferred to a heparinized-tube and inverted 10 times for adequate heparin coating to prevent coagulation. Samples were retained at room temperature (RT) for 30 min prior to centrifugation at RT for 10 min at 1300 g (Zhao et al., 2018). Plasma was collected, aliquoted for each biochemical assay, and stored at −80°C until further analyses. Rats were supplemented with 0.5 ml saline after each blood draw.

### Assessment of Locomotor Function

The λ-carrageenan-induced locomotor activity impairment (CLAIM) model was employed as a measure of non-evoked inflammatory nociceptive behavior (Zhu et al., 2012) to increase clinical translatability relative to traditional models of evoked mechanical hypersensitivity (e.g., von Frey filament test). Locomotor activity was monitored and quantified using an open field photobeam activity system (San Diego Instruments, San Diego, CA, United States). Clear Plexiglas chambers (40 × 40 × 40 cm) were surrounded by a 16 × 16 photobeam matrix positioned 4 cm from chamber floor. Consecutive photobeam breaks within the central 30 × 30 cm of the activity monitor were recorded as central ambulation. Peripheral ambulation was counted as consecutive beam breaks in the surrounding perimeter. Central and peripheral ambulations were summed to provide a measure of total horizontal ambulation. Vertical activity was recorded using a row of 16 photobeams, positioned 16 cm from the activity monitor floor; breaks in these beams indicated vertical (rearing) activity. Catheterized rats (*n* = 12) were habituated to the activity monitors for three 30-min intervals, aligning with the timepoints recorded on the experimental day. The following day, rats were returned to the activity chambers for baseline assessment. Rats were injected with i.p.l. saline or λ-carrageenan 24 h later and locomotor activity was assessed at four later timepoints (1, 5, 8, and 24 h); each activity session lasted 30 min. Prior to baseline and experimental sessions, catheter blood was collected, and paw thickness recorded as described above.

### Quantification of Cytokine Expression

Plasma levels of 23 cytokines [G-CSF, GM-CSF, GRO/KC (CXCL1), IFN-γ, IL-1α, IL-1β, IL-2, IL-4, IL-5, IL-6, IL-7, IL-10, IL-12 (p70), IL-13, IL-17A, IL-18, M-CSF, MCP-1 (CCL2), MIP-1α, MIP-3α, RANTES, TNF-α, and VEGF] were measured using Bio-Plex Pro Rat Cytokine 23-Plex™ (#12005641) magnetic bead-based assays (Bio-Rad Laboratories) on the Bio-Plex^®^ platform (Bio-Rad), according to the manufacturer’s instructions. Plasma samples were assayed at a dilution of 1:4 and run in triplicate. Cytokine expression (pg/ml) for each sample was multiplied by the dilution factor prior to percent baseline calculation.

### Synthesis of Nanobiosensors

Detailed descriptions of the syntheses of nanobiosensors, the required components and all required processes are discussed in detail in our previous studies (Wang et al., 2014; Udukala et al., 2016). Briefly (Figure 1B), nanobiosensors were assembled from dopamine coated Fe/Fe_3_O_4_ nanoparticles, cyanine 5.5, and consensus sequences for MMP2 (GAGIPVS-LRSGAG) and MMP9 (GAGVPLS-LYSGAG) that were linked to TCPP on resin (Wang et al., 2014). To achieve this synthesis, a solution was prepared by completely dissolving 64 mg of the TCPP-linked peptide sequence, 37 mg of Cy 5.5, 45 mg of EDC and 45 mg of DMAP in 30 ml of anhydrous dimethylformamide (DMF). In a second vial, 450 mg of dopamine-coated Fe/Fe_3_O_4_ nanoparticles were dispersed in 10 ml of anhydrous DMF by sonicating for 20 min. Both solutions were then mixed, sonicated for 10 min, and incubated overnight in a shaker at RT. After overnight incubation, the resulting nanobiosensor was collected *via* centrifugation (5 min at 10,000 RPM), washed with DMF to removed excess dye and unbound components, followed by five washes with cold ether (−10°C). After each washing step, the nanobiosensor was collected *via* centrifugation. The nanobiosensor was then collected, dried with argon gas, and stored at −20°C.

### Calibration and Proteolytic Activity Measurements for Matrix Metalloproteinase 2 and Matrix Metalloproteinase 9

HEPES-buffer (25 µM) was enriched with Ca(II), Mg(II), and Zn(II)-enriched (10 µM each) HEPES buffer {2-[4-(2-hydroxyethyl) piperazin-1-yl] ethanesulfonic acid} (pH = 7.2). Detection of nanobiosensor fluorescence (λexc = 421 ± 10 nm, λem: 650 ± 20 nm) was performed utilizing a 96-well fluorescence plate reader (BioTek Synergy H1). Proteolytically active, recombinant MMPs were purchased from Enzo Lifesciences (Farmingdale, NY) for calibration. Stock solutions (1 µM in HEPES) were prepared for each enzyme or cytokine/chemokine and then diluted to specification with HEPES (Udukala et al., 2016; Kalubowilage et al., 2018). The nanobiosensors for MMP2 and MMP9 were dispersed (0.30 mg each) in 1.0 ml of HEPES buffer by means of sonication for 10 min at 25°C. After the sonication procedure, the nanobiosensor dispersions are usable for 1 h and can be re-dispersed again by sonication.

Three types of dispersion solutions were prepared for calibration and proteolytic activity measurements in the 96-well plate and incubated for 60 min at 26°C: (A) Sample Control: 125 µl HEPES (as described above) plus 5 µl HEPES containing a defined amount of the selected enzyme or 5 µl of collected plasma sample; (B) Assay Control: 125 µl of assay (nanobiosensors dispersed in HEPES) plus 5 µl HEPES; (C) Assay: 125 µl of assay plus 5 µl HEPES containing a defined amount of the selected enzyme or 5 µl of collected plasma sample.

### Statistical Analyses

The timecourse of inflammation (paw thickness), locomotor activity, and biomarker expression was normalized to baseline measurements prior to saline- or λ-carrageenan treatment. We normalized the activity of MMP9 to MMP2 as MMP2 has been shown to participate in the late phase of the inflammatory response (≥24 h) (Kawasaki et al., 2008; Nascimento et al., 2013), thus serving as an internal control in the acute timecourse presented. The impact of λ-carrageenan treatment on the timecourse of paw inflammation, locomotor activity, cytokine expression, and MMP activity were analyzed using a two-way repeated measures analysis of variance (ANOVA) with the factors of time (1–24 h) and treatment (saline or λ-carrageenan) followed by Sidak’s preplanned comparisons analysis, as appropriate. The relationships between paw inflammation, locomotor activity, cytokine expression, and MMP activity were determined using Pearson’s correlations analyses. Analyses were preformed using GraphPad Prism (version 9.2.0).

## Results

### Intraplantar Administration of λ-Carrageenan Induces Acute Inflammation and Non-Evoked Inflammatory Nociceptive Behavior

**FIGURE 2:**
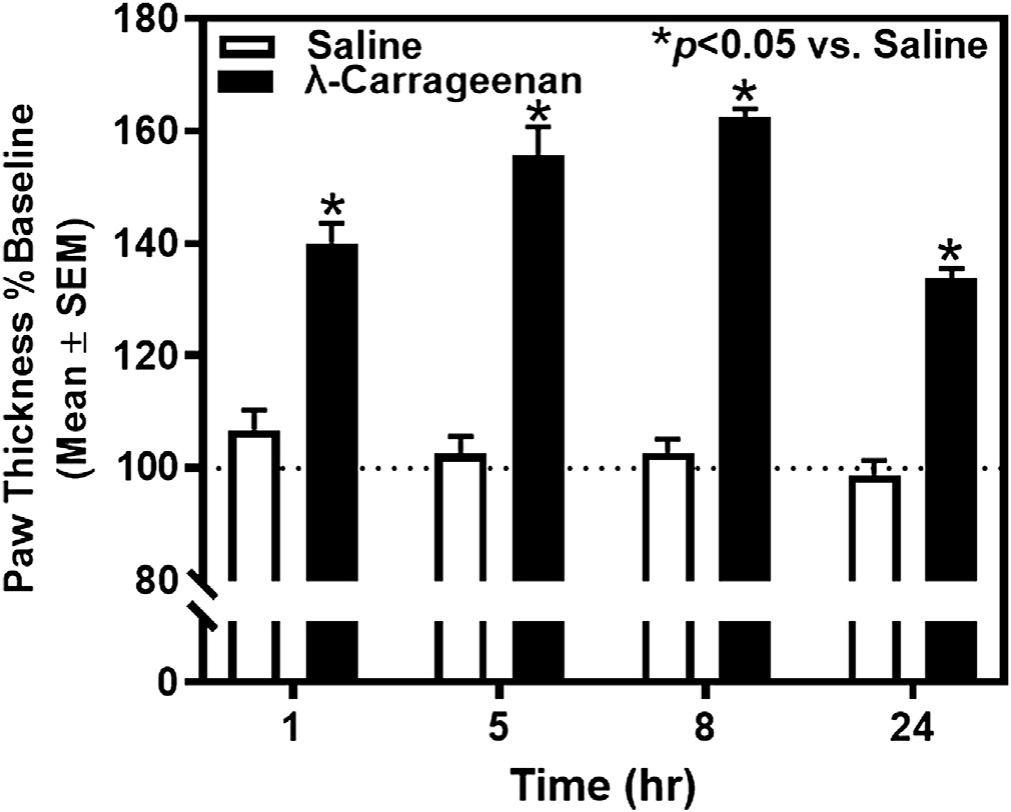
The effect of λ-carrageenan (1%; i.p.l.) on paw thickness across 24 h is presented. The percent baseline paw thickness was calculated as the mean value at each time point following treatment with saline (white bars) or λ-carrageenan (black bars), normalized to baseline values. λ-Carrageenan significantly increased paw thickness relative to saline at all time points (*p < 0.05 vs. Saline).

We tested the hypothesis that i.p.l. treatment with λ-carrageenan will induce paw inflammation and impair mobility assessed by paw thickness and locomotor activity counts, respectively. Baseline paw thickness for each rat was determined 24 h prior to footpad injection with saline or λ-carrageenan and used to calculate percent baseline values of paw thickness 1–24 h following treatment (Figure 2). Across the 24 h, there was a main effect of time (F_3,30_ = 18.69, *p* < 0.05) and treatment (F_1,10_ = 179.60, *p* < 0.05) and significant time × treatment interaction (F_3,30_ = 15.75, *p* < 0.05) on paw thickness. We found that injection of λ-carrageenan significantly increased paw thickness at all timepoints considered.

**FIGURE 3:**
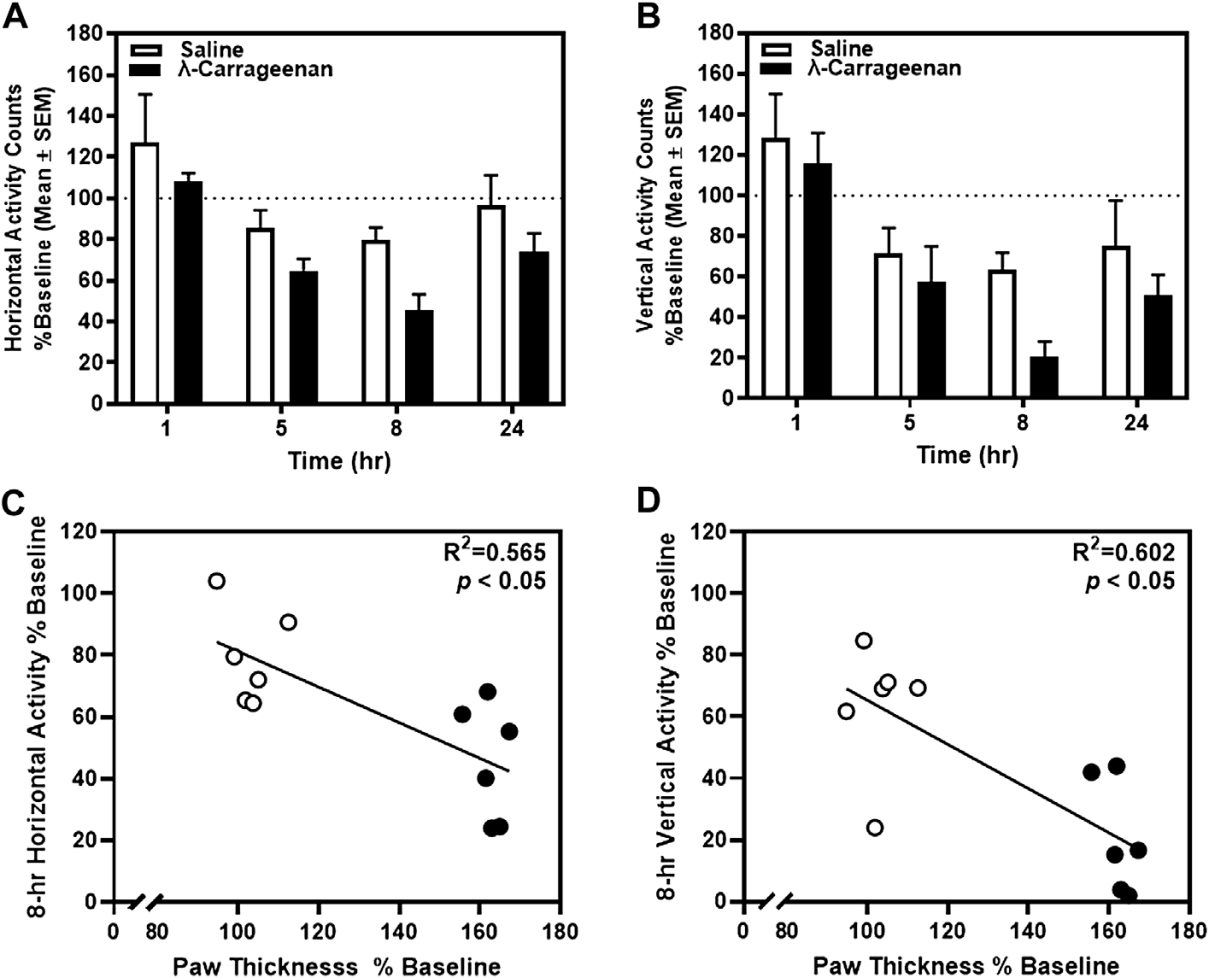
The effect of λ-carrageenan (1%; i.p.l.) on locomotor activity is presented. Percent baseline horizontal and vertical activity for individual rats was calculated as the mean value at each time point following treatment with saline (white bars) or λ-carrageenan (black bars), normalized to baseline value. The timecourses of horizontal **(A)** and vertical **(B)** activity are presented. Paw thickness negatively correlated with horizontal **(C)** and vertical **(D)** activity at the 8 h timepoint.

We next determined the impact of λ-carrageenan on mobility by quantifying horizontal and vertical activity counts for each rat in locomotor chambers. Baseline horizontal and vertical activity was determined 24 h prior to footpad injection with saline or λ-carrageenan and used to calculate percent baseline values of these activity measures 1–24 h following treatment (Figures 3A,B). A main effect of time on horizontal (F_3,30_ = 16.99, *p* < 0.05) and vertical activity (F_3,30_ = 12.76, *p* < 0.05) was detected, but no main effect of treatment (horizontal, F_1,10_ = 3.37, *p >* 0.05; vertical, F_1,10_ = 2.91, *p >* 0.05) or time × treatment interaction (horizontal, F_3,30_ = 0.35, *p >* 0.05; vertical F_3,30_ = 0.52, *p >* 0.05). Pearson’s correlation analyses revealed a significant negative correlation between horizontal activity and paw thickness (R^2^ = 0.565, *p* < 0.05) (Figure 3C), as well as vertical activity and paw thickness (R^2^ = 0.602, *p* < 0.05) at the 8 h timepoint (Figure 3D). No additional correlations were detected at other time points.

### Increased Plasma Levels of C-X-C Motif Chemokine Ligand 1 and C-C Motif Chemokine Ligand 2 Associate With Peak Measures of Reduced Mobility and Inflammation

**FIGURE 4:**
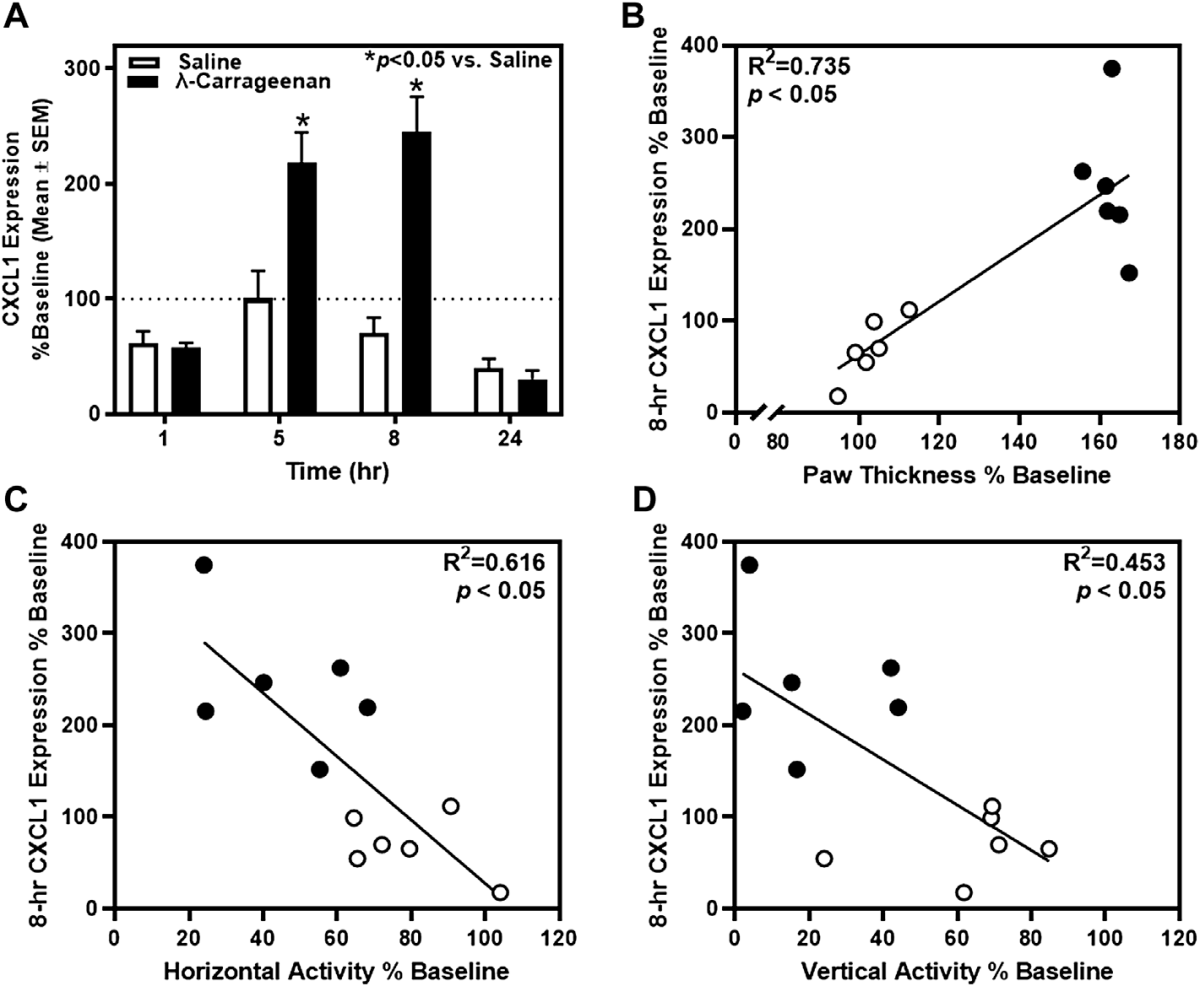
The impact of λ-carrageenan on the timecourse of CXCL1 expression is presented with correlational analyses. **(A)** The percent baseline CXCL1 expression was calculated as the mean value at each time point following treatment with saline (white bars) or λ-carrageenan (black bars), normalized to baseline values. λ-Carrageenan significantly increased CXCL1 expression relative to saline at 5 and 8 h (*p < 0.05 vs. saline). **(B)** CXCL1 expression positively correlated with paw thickness and negatively correlated with **(C)** horizontal and **(D)** vertical activity at the 8 h timepoint.

To identify specific onset and offset of inflammatory biomarkers detectable in plasma, we collected plasma from rats at baseline and each subsequent time point using the Bio-Plex Pro Rat Cytokine 23-Plex assay which assesses 23 cytokines. Of these 23 targets, we detected CXCL1, CCL2, MIP-1α, MIP-3α, and RANTES. We detected a main effect of treatment (F_1,10_ = 18.31, *p* < 0.05) and time (F_3,28_ = 27.37, *p* < 0.05) for CXCL1 expression, as well as a significant treatment × time interaction (F_3,28_ = 13.98, *p* < 0.05) (Figure 4A). Specifically, a significant increase in CXCL1 expression was observed in plasma samples from λ-carrageenan-injected rats, relative to saline-treated rats, at the 5 and 8 h timepoints. These effects were completely normalized at the 24 h timepoint. As anticipated, a positive correlation between CXCL1 expression and paw thickness was observed (R^2^ = 0.735, *p* < 0.05) (Figure 4B). A negative correlation between CXCL1 expression and both horizontal activity (R^2^ = 0.616, *p* < 0.05) and vertical activity (R^2^ = 0.453, *p* < 0.05) was seen at the 8 h timepoint (Figures 4C,D).

**FIGURE 5:**
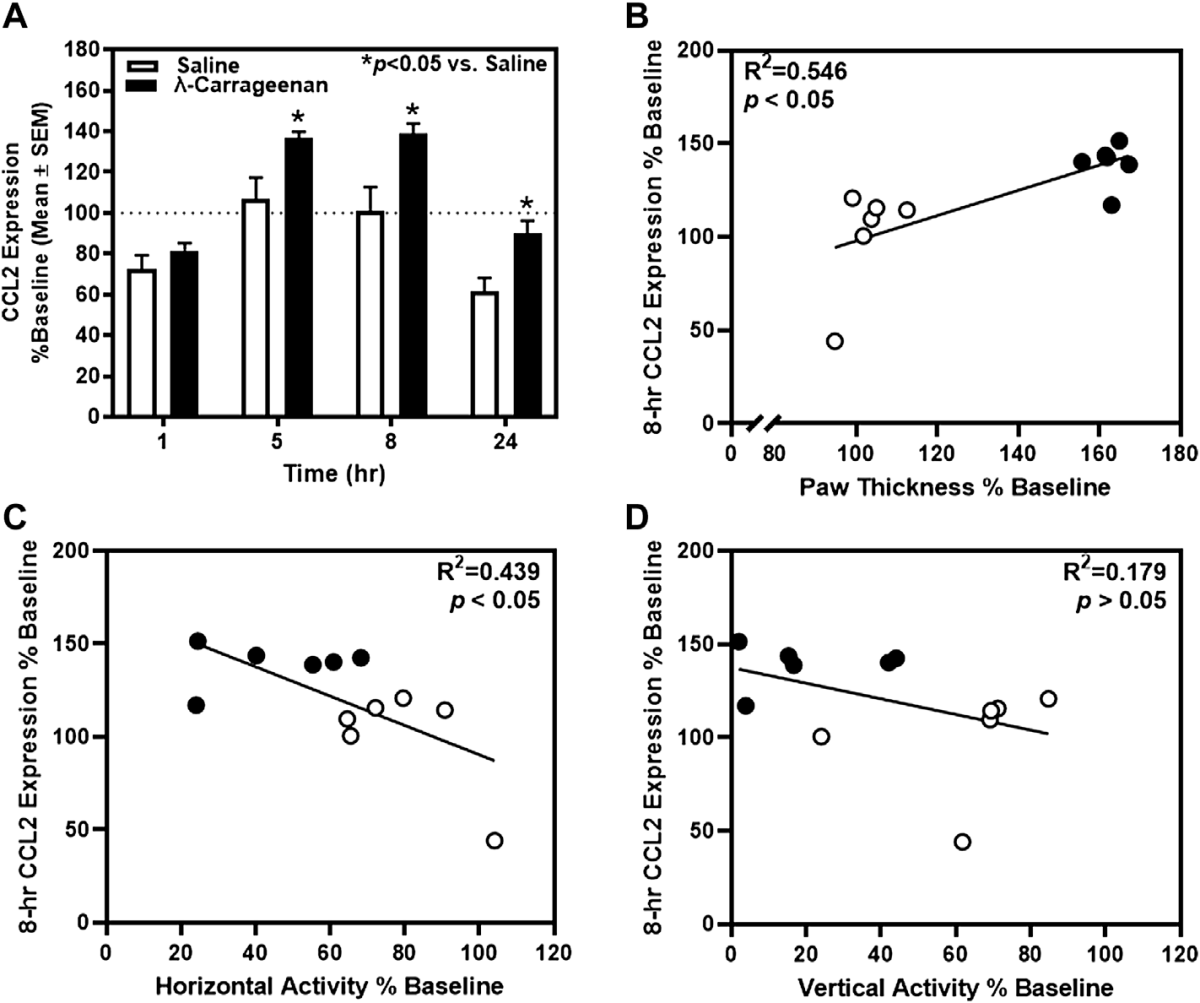
The impact of λ-carrageenan on the timecourse of CCL2 expression is presented with correlational analyses. **(A)** The percent baseline CCL2 expression was calculated as the mean value at each time point following treatment with saline (white bars) or λ-carrageenan (black bars), normalized to baseline values. λ-Carrageenan significantly increased CCL2 expression relative to saline at the 8 and 24 h timepoints (*p < 0.05 vs. Saline). CCL2 expression **(B)** positively correlated with paw thickness and **(C)** negatively correlated with horizontal activity at the 8 h timepoint. **(D)** No significant correlation between CCL2 expression and vertical activity was detected.

For CCL2, a main effect of treatment (F_1,10_ = 7.98, *p* < 0.05) and time (F_3,30_ = 119.3, *p* < 0.05), and a treatment × time interaction (F_3,30_ = 6.898, *p* < 0.05) were observed (Figure 5A). Significant increases in CCL2 expression were detected at the 5, 8, and 24 h timepoints. At the 8 h timepoint, a positive correlation between CCL2 expression and paw thickness (R^2^ = 0.546, *p* < 0.05) (Figure 5B) and a negative correlation between CCL2 expression and horizontal activity (R^2^ = 0.439, *p* < 0.05) were detected; CCL2 expression did not relate to vertical activity at this timepoint (R^2^ = 0.179, *p* > 0.05) (Figures 5C,D). There was no main effect of treatment or significant treatment × time interaction observed for the additional cytokines detected in the Bio-Plex assay.

### Heightened Matrix Metalloproteinase 9/Matrix Metalloproteinase 2 Activity Serves as an Early Predictor of Non-Evoked Inflammatory Nociceptive Behavior

**FIGURE 6:**
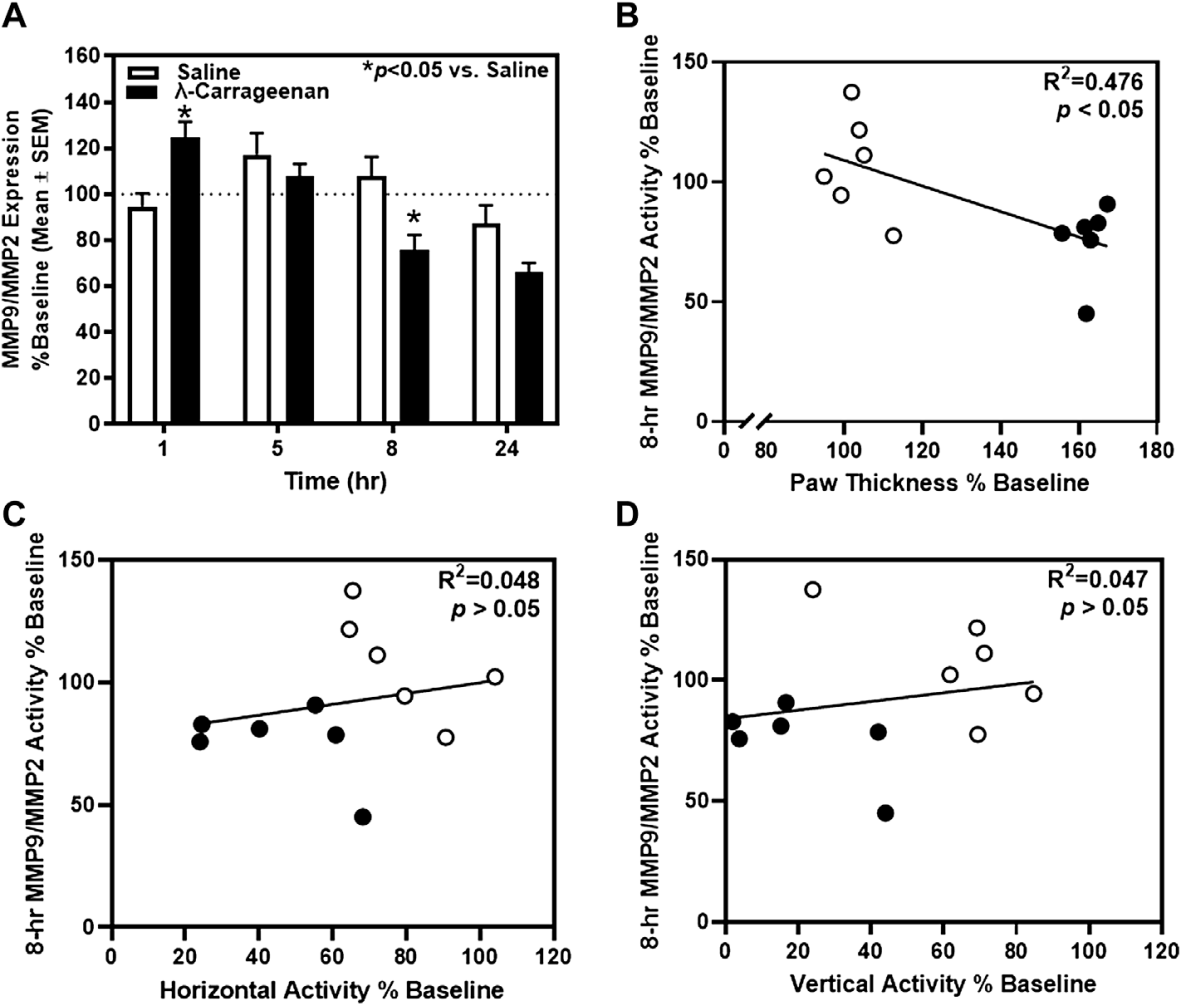
The impact of λ-carrageenan on the timecourse of MMP9/MMP2 activity is presented with correlational analyses. The percent of baseline MMP9/MMP2 activity was calculated as the mean value at each time point following treatment with saline (white bars) or λ-carrageenan (black bars), normalized to baseline values. **(A)** λ-Carrageenan significantly increased MMP9/MMP2 activity at the 1 h, and decreased MMP9/MMP2 activity, at the 8 h timepoint, respectively (*p < 0.05 vs. Saline). **(B)** MMP9/MMP2 activity negatively correlated with paw thickness at the 8 h timepoint. No significant correlation between MMP9/MMP2 activity and locomotor activity was detected **(C,D)**.

We employed novel, ultrasensitive fluorescence nanobiosensors to detect fluctuations in MMP9 and MMP2 activity observable in the plasma of saline- and λ-carrageenan-treated rats. The timecourse analysis of MMP9/MMP2 activity identified a main effect of treatment (F_1,10_ = 4.462, *p* = 0.06) and a main effect of time (F_3,30_ = 10.22, *p* < 0.05), and a significant treatment × time interaction (F_3,30_ = 6.593, *p* < 0.05) (Figure 6A). MMP9/MMP2 activity was increased and decreased at the 1 and 8 h timepoints in rats treated with λ-carrageenan, relative to saline. Pearson’s correlation analyses revealed a negative correlation between MMP9/MMP2 activity and paw thickness at the 8 h timepoint (R^2^ = 0.476, *p* < 0.05) (Figure 6B), but no correlation with locomotor activity at this timepoint (horizontal, R^2^ = 0.048, *p* > 0.05; vertical, R^2^ = 0.047, *p* > 0.05) (Figures 6C,D).

## Discussion

The primary goal of the current study was to explore the feasibility of utilizing a robust sensing platform with liquid biopsy techniques to quantify biomarkers of inflammatory nociception. Specifically, we monitored the kinetic activity profile of detectable cytokines and MMP9/MMP2 ratio in the plasma of rats experiencing acute inflammation and identified correlations between these fluctuations and paw edema, as well as non-evoked inflammatory nociceptive behavior. To the best of our knowledge, no previous study has surveyed the timecourse of cytokine protein concentrations or MMP activity in plasma in a rodent model of acute inflammation. Paw thickness was significantly greater in λ-carrageenan-injected rats versus saline-injected rats at all time points, with peak inflammation observed the 8 h timepoint after treatment. While we did not detect a main effect of treatment on the timecourse of locomotor activity, we did detect a strong negative correlation between paw thickness and locomotor activity at 8 h after intraplantar injection. These data suggest the existence of a threshold of inflammation that must be surpassed to adversely impact mobility. We then narrowed subsequent correlational analyses between cytokine expression/MMP activity and paw inflammation/locomotor activity to the 8 h time point.

Cytokines CXCL1 and CCL2 are small peptide chemoattractants responsible for recruiting several classes of immune cells (i.e., monocytes, neutrophils, etc.) to the site of injury. Average plasma CXCL1 protein expression dramatically increased in λ-carrageenan-injected rats, relative to saline, at the 5 and 8 h timepoints after treatment. Further, greater CXCL1 expression at the 8 h timepoint was associated with increased paw thickness and reduced locomotor activity. These data agree with a previous study demonstrating increased *Cxcl1* mRNA expression in inflamed tissue following intraplantar λ-carrageenan treatment (Goto et al., 2021). Increases in *Cxcl1* mRNA were greatest 1–4 h post-treatment, whereas differences in plasma CXCL1 protein expression were most pronounced at 5–8 h in the current study. This discrepancy may be explained by the sequential orchestration of transcription and translation (Crick, 1958). Moreover, our ability to detect inflammatory biomarkers and the timecourse of their expression is undoubtedly dependent on the source of the sample (localized inflamed tissue versus circulating blood). Modest, yet significant, increases in CCL2 protein expression were seen at the eight and 24 h time points in the current study. Similar to CXCL1, we found increased CCL2 expression associates with heightened paw inflammation and reduced locomotor activity at the 8 h timepoint. Not surprisingly, *Ccl2* mRNA expression was also increased in the λ-carrageenan-injected paw sooner than we detected the increases in plasma protein expression (Goto et al., 2021). We saw a trending increase in CCL2 plasma expression 5 h post treatment, in line with previous research reporting a significant increase in CCL2 serum expression 4 h later after intraplantar λ-carrageenan administration (Ou et al., 2019). In contrast to the present findings, Ou et al. (2019) reported significant changes in serum protein expression of IL-1β at 4 h post-treatment, whereas there was no main effect of carrageenan-injection on any of the other three cytokines detected (MIP-1α, MIP-3α, and RANTES; data not shown). This divergence might be explained by differences in the timing of blood draws for liquid biopsy as our timecourse analyses display distinct, time-dependent variation in cytokine expression.

The onset of MMP2 activity is delayed following tissue damage and thus was used as an internal calibration of MMP9 activity in our acute model of inflammatory nociception (Kawasaki et al., 2008; Nascimento et al., 2013). We observed the expected rapid and transient induction of MMP9/MMP2 activity at the 1 h timepoint in λ-carrageenan-injected rats, relative to saline-injected rats. This effect was reversed at the 8 h timepoint. Previous studies investigated the regulatory role of MMP inhibition on cytokine and chemokine expression through use of broad-spectrum MMP inhibitors [i.e., doxycycline, GM6001 (Galardin^®^)] (Supasorn et al., 2016; Tang et al., 2017). Tang and colleagues found that pan-MMP inhibition resulted in a decrease in tissue expression of CXCL1 and CCL2 (Supasorn et al., 2016). However, at the height of peak inflammation in the current study, MMP9/MMP2 activity is significantly lower in λ-carrageenan-injected animals while CXCL1 and CCL2 expression is increased. Future studies will be designed to disentangle the role of each functional class of MMPs [e.g., collagenases (MMP1), gelatinases (MMP9, MMP2)] on cytokine/chemokine induction with more selective pharmacological compounds. It should also be mentioned that this is the first study in which the proteolytic activity of MMP2 and MMP9 was measured, not simply the protease concentrations.

The present studies were conducted in male rats and given sex-specific distinctions in production of cytokines and the overall immune response (Klein and Flanagan, 2016), future studies are necessary to determine if the temporal pattern of biomarker expression and activity is generalizable to females. The Bio-Plex assay detected a limited number of cytokines across all timepoints in both saline- and λ-carrageenan-injected rats (six of 23 assayed). It is probable that the expression of circulating cytokines in plasma is much lower in comparison to the localized site of inflammation. Nonetheless, our ability to capture differences in plasma CXCL1 and CCL2 expression suggests these cytokines will be useful as reliable markers of inflammatory nociception in liquid biopsies.

We provide foundational data for identifying quantitative markers of inflammatory nociception in liquid biopsies of inflammatory pain patients. We propose that CXCL1 shows the most promise as a readily detected peripheral biomarker of nociceptive inflammation as we observed robust changes in carrageenan-injected animals. Further, a recent clinical trial observed a significant reduction in serum CXCL1 protein expression following treatment with an anti-inflammatory agent in rheumatoid arthritis patients (Tao et al., 2021). This observation coincided with reduced clinical symptoms of pain in these patients. Relative changes observed in CCL2 expression and MMP9/MMP2 activity in the current study were modest. Previous clinical trials identified moderate changes in potential biomarkers of pain *via* the liquid biopsy approach (i.e., cystatin C in cerebrospinal fluid of women in active labor) (Mannes et al., 2003). However, cystatin C was later found to be an unreliable marker of pain in a follow-up study with a greater number of participants; cystatin C expression also did not correlate with pain levels of neuropathic conditions (Eisenach et al., 2004; Kalso, 2004). Future studies are required to expand on the current findings by identifying robust biosignatures of inflammatory regulators unique to various origins of pain (e.g., neuropathic) and how they might differ across species and sex. Our ultimate goal is to develop improved diagnostic POC devices to objectively quantify inflammatory nociception with increased reliability, relative to current the current standard-of-care practices that rely on subjective measures of self-report (Younger et al., 2009). The successful development of POC devices will enable health care providers in clinical settings to guide appropriate treatment strategies and optimize management of pain patients.

## Data Availability

The original contributions presented in the study are included in the article/supplementary material, further inquiries can be directed to the corresponding author.

## Abbreviations

ANOVA, analysis of variance; CLAIM, carrageenan-induced locomotor activity impairment; Cy5.5, cyanine 5.5; DMF, dimethylformamide; FRET, Foerster Resonance energy-transfer; HEAL, Helping to End Addiction Long-Term; i.p.l., intraplantar; MMP, matrix metalloproteinases; POC, point-of-care; RT, room temperature; TCPP, tetrakis-carboxyphenyl-porphyrin.
